# Metastatic Pulmonary Calcification in Multiple Myeloma in a 45-Year-Old Man

**DOI:** 10.1155/2013/341872

**Published:** 2013-04-11

**Authors:** Salim R. Surani, Sara Surani, Amina Khimani, Joseph Varon

**Affiliations:** ^1^Department of Medicine, Texas A&M University, 7613 Lake Bolsena, Corpus Christi, TX 78413, USA; ^2^Pulmonary Associates of Corpus Christi, 1177 Wheeler Avenue, Aransas Pass, TX 78366, USA; ^3^Department of Acute and Continuing Care, The University of Texas Health Science Center, Houston TX 77030, USA

## Abstract

Metastatic calcification has been associated with multiple-myeloma-induced hypercalcemia. Despite of a relatively high prevalence of metastatic pulmonary calcification in patients with multiple myeloma, only a few cases have been clinically and radiologically detected. A 45-year-old Hispanic male presented to the Emergency Department with complaint of worsening weakness and myalgia. Laboratory findings revealed renal insufficiency and hypercalcemia. CT scan of chest revealed calcified pleural and pulmonary nodule. Technetium (Tc) 99 bone scan revealed diffuse activity in the pulmonary parenchyma consistent with metastatic pulmonary calcification. Metastatic pulmonary calcification, despite its high prevalence, remains undetected. This is, in part, due to its radiographic characteristic properties that evade detection by routine imaging studies. We present a case of a metastatic pulmonary calcification in a patient diagnosed with multiple myeloma and chronic kidney disease, as well as a brief literature review including clinical findings and treatment options.

## 1. Introduction

Metastatic pulmonary calcification (MPC) is a common complication of multiple myeloma (MM) and it is result of the high levels of calcium-phosphate products deposited in alveolar and vessel walls of normal lung [1]. It is known to be aggravated by physical stress or tissue injury, which clinically manifests as progressive dyspnea, hypoxemia, and worsening symptoms of respiratory insufficiency and findings of restrictive pulmonary function test [2]. Both benign and malignant oncological etiologies can cause MPC. Some of the “benign” causes include excess exogenous administration of calcium and vitamin D, hyperparathyroidism, hypervitaminosis D, chronic renal insufficiency, osteoporosis, and *osteitis deformans*. Malignant etiologies include multiple myeloma, parathyroid carcinoma, leukemia, lymphoma, breast carcinoma, synovial carcinoma, choriocarcinoma, and hypopharyngeal squamous carcinoma. 

In metastatic calcification, common sites of calcium deposits involve lung, kidney, gastric mucosa, heart, and blood vessels. Histopathologically, MPC involves diffuse calcium deposition in the lung with deposits typically in alveolar septa, bronchi, pulmonary vessels, and myocardium [1]. The composition of the deposits varies, with lung and soft tissue tumors primarily having a hydroxyapatite calcification while the renal failure calcifications present [3]. Based on the calcium deposition in the lungs three patterns have been identified: multiple diffuse calcified nodules (as seen in our patient), apical or basal calcified nodules, and parenchymal or lobar consolidation and calcified nodules [4, 5].

## 2. Case Presentation

**Figure 1:**
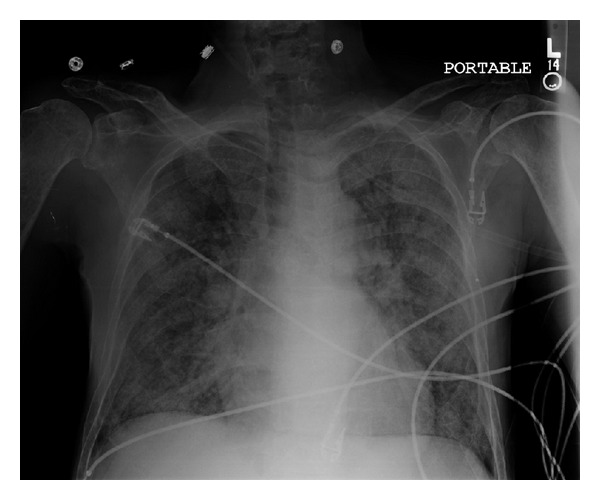
Chest X-ray AP view showing reticulonodular infiltrates and microcalcifications.

**Figure 2:**
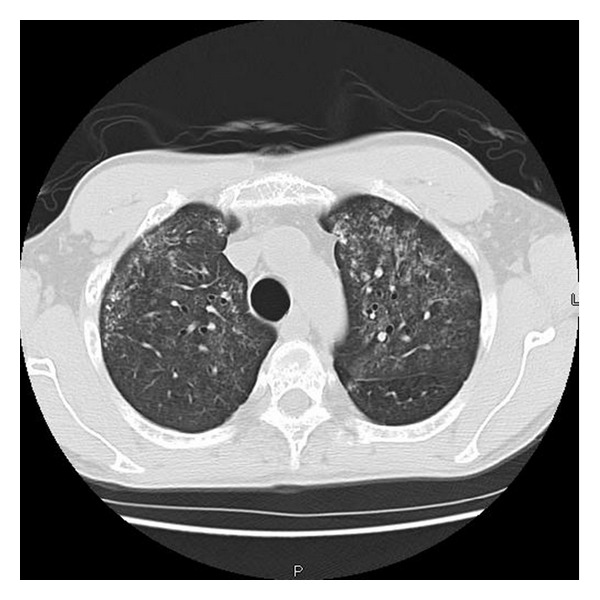
Chest CT showing ground-glass nodular opacities, with poorly defined nodules.

**Figure 3:**
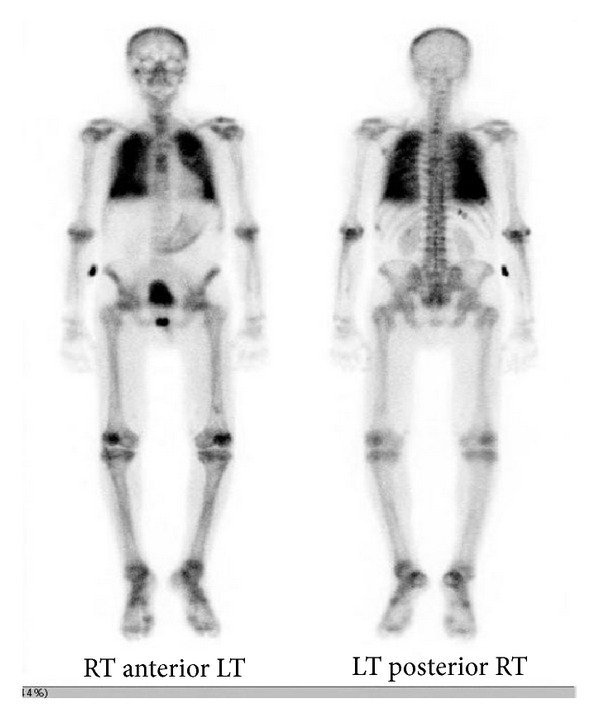
Technetium 99 bone scan, which revealed diffuse activity in the lung with significant lighting up of pulmonary parenchyma.

A 45 year-old Hispanic male presented to the emergency department (ED) with complaints of weakness and myalgias for the preceding month with worsening on the days prior to admission. Physical exam was grossly unremarkable and vital signs were stable. He was found to be anemic with hemoglobin of 7.8 g/dL. In addition he had a platelet count of 73,000/mL. Other pertinent laboratory findings included a blood urea nitrogen (BUN) of 41 mg/dL, creatinine of 2.6 mg/dL, calcium 12.3 mg/dL and ionized calcium level was 1.59. Total protein was 7.6 g/dL and albumin was 4.1 g/dL. The patient's chest radiograph showed increased vascular markings and reticulonodular opacifications (see Figure 1). Computed tomography (CT) scans of chest also revealed diffuse ground-glass nodular opacities, with numerous poorly defined nodules measuring 3–10 mm in diameter both (see Figure 2). A skeletal survey revealed punched-out defects consistent with multiple myeloma (MM). The patient underwent a Technetium (Tc) 99 bone scan, which revealed diffuse activity in the lung with significant lighting up of the pulmonary parenchyma, consistent with metastatic pulmonary calcification (see Figure 3). 

A subsequent serum protein electrophoresis revealed elevation of the free lambda light chain to 14,400 mg/L (normal range 5.71–26.3 mg/L). A bone marrow biopsy revealed massive plasmacytosis. The patient underwent standard therapy for his anemia and hypercalcemia and was seen by the oncology and nephrology services. Once discharged the patient was eventually lost in followup due to medical noncompliance. Our last search indicated that the patient has expired due to hypoxia and noncompliance with the hemodialysis sessions.

## 3. Discussion

Despite its common occurrence in MM, MPC is rarely detected due to the poor sensitivity for antemortem diagnosis by chest radiography and other conventional investigations. In a study by Kintzer and associates, only 10% of patients with multiple myeloma were detected with pulmonary calcifications [6]. Mediastinal computed tomography (CT) and high-resolution CT (HRCT) have high sensitivity in detecting smaller calcifications, thus proven to be one of the best diagnostic investigations, thus decreasing the need for open lung biopsy in this condition [4, 5]. The other useful technique is nuclear imaging using Tc-99 as has been done in our patient, wherein the lungs affected by PMC demonstrate an increase uptake of the radioactive isotope. Dual energy chest radiography has higher sensitivity in detecting PMC when compared to routine chest radiography [7]. In addition Magnetic Resonance Imaging (MRI) has been used. 

Treatment of this condition is primarily symptomatic, which includes lowering of calcium-phosphate products in the body with use of phosphate binders. Isolated hyperphosphatemia and tertiary hyperparathyroidism also may be treated with phosphate binders. Prompt management of secondary and tertiary hyperparathyroidism is necessary to avoid uncontrolled extraskeletal calcification, ischemic skin necrosis, pruritis, and hyperparathyroid bone disease [8]. Prompt therapy with calcium and vitamin D supplements is initiated and if unresponsive to medical therapy, parathyroidectomy is indicated.

## 4. Conclusion

MPC is an asymptomatic condition, which may remain undiagnosed and untreated, progressing to irreversible lung damage and respiratory failure. Hence, early detection, prompt treatment, and research to develop new diagnostic and treatment modalities gain center stage for enhancing the management of this condition.
